# What do external consultants from private and not-for-profit companies offer healthcare commissioners? A qualitative study of knowledge exchange

**DOI:** 10.1136/bmjopen-2014-006558

**Published:** 2015-02-05

**Authors:** Lesley Wye, Emer Brangan, Ailsa Cameron, John Gabbay, Jonathan H Klein, Rachel Anthwal, Catherine Pope

**Affiliations:** 1 School of Social and Community Medicine, University of Bristol, Bristol, UK; 2 School of Policy Studies, University of Bristol, Bristol, UK; 3 Wessex Institute for Health Research and Development, University of Southampton, Southampton, UK; 4 Southampton Business School, University of Southampton, Southampton, UK; 5 South West Commissioning Support Unit, Bristol, UK; 6 Faculty of Health Sciences, Southampton, UK

## Abstract

**Objectives:**

The use of external consultants from private and not-for-profit providers in the National Health Service (NHS) is intended to improve the quality of commissioning. The aim of this study was to learn about the support offered to healthcare commissioners, how external consultants and their clients work together and the perceived impact on the quality of commissioning.

**Setting:**

NHS commissioning organisations and private and not-for-profit providers.

**Design:**

Mixed methods case study of eight cases.

**Data collection:**

92 interviews with external consultants (n=36), their clients (n=47) and others (n=9). Observation of 25 training events and meetings. Documentation, for example, meeting minutes and reports.

**Analysis:**

Constant comparison. Data were coded, summarised and analysed by the research team with a coding framework to facilitate cross-case comparison.

**Results:**

In the four contracts presented here, external providers offered technical solutions (eg, software tools), outsourcing and expertise including project management, data interpretation and brokering relationships with experts. In assessing perceived impact on quality of commissioning, two contracts had limited value, one had short-term benefits and one provided short and longer term benefits. Contracts with commissioners actively learning, embedding and applying new skills were more valued. Other elements of success were: (1) addressing clearly agreed problems of relevance to managerial *and* operational staff (2) solutions co-produced at all organisational levels (3) external consultants working directly with clients to interpret data outputs to inform locally contextualised commissioning strategies. Without explicit knowledge exchange strategies, outsourcing commissioning to external providers resulted in the NHS clients becoming dependent.

**Conclusions:**

NHS commissioning will be disadvantaged if commissioners both fail to learn in the short term from the knowledge of external providers and in the longer term lose local skills. Knowledge exchange mechanisms are a vital component of commissioning and should be embedded in external provider contracts.

**Strengths and limitations of this study**

- This is the largest study of the use of external consultants to support healthcare commissioners in England post-Health and Social Care Act 2012.
- This study illuminates the potential benefits and challenges in using external expertise in commissioning.
- Case study results can offer substantial, information rich accounts of the role of external consultants and assess their perceived impact on commissioning decisions. But case studies cannot assess the actual impact on commissioning and are not statistically generalisable. However, findings are transferable to similar settings.
- Perhaps because the research team were overly associated with external consultants as this was the access point in fieldwork, we obtained fewer accounts from the National Health Service (NHS) clients. Recruiting another not-for-profit agency would have further augmented comparative analyses.
- This study emphasises the importance of taking steps to improve knowledge exchange between external consultants and their NHS clients to gain the greatest benefit from these types of contracts.

## Introduction

Healthcare commissioners plan services and allocate funding to meet the needs of specific populations in England. Over two decades, commissioning (or ‘purchasing’ as it was originally known) has taken different organisational forms, including Health Authorities, Primary Care Groups, Primary Care Trusts (PCTs) and now Clinical Commissioning Groups (CCGs). There are many definitions of ‘commissioning’.^1^ An early, simple conceptual framework by Øvretveit^2^ used the Plan-Do-Study-Act model to illustrate commissioning activities. The Department of Health has developed a more complicated model which includes assessing needs, designing services and managing demand and performance.^3^ Gradually, commissioning has become both more complex and better understood.

A growing body of evidence suggests that commissioning is “messy, fragmented and largely accomplished in meetings”^4^ with progress made through “bite sized pieces of work”.^5^ Moreover, commissioning is challenging and difficult to do well, regardless of whether the healthcare system is English, European or American.^6^ In 2007, World Class Commissioning was introduced in the National Health Service (NHS)^7^ along with FESC (Framework for Procuring External Services for Commissioners) which authorised commercial providers to work with commissioners.^8^ With the advent of the Coalition government in 2010, FESC was dissolved but the Lead Provider framework for commercial and other external providers such as commissioning support units has taken its place.^9^ The assumption with all these initiatives is that use of external providers will lead to higher quality commissioning.^8^ ^10^ However, despite an estimated £308.5 million received by external management consultants from the NHS in 2007–2008,^11^ there remains scepticism about the benefits of using these services. For example, a *Health Service Journal* survey of 93 senior commissioning directors found that nearly half thought that commercial consultants would make only a ‘little’ difference.^8^

Nonetheless, the terrain for external provision in the NHS has become even more favourable. The White Paper *Liberating the NHS*^12^ and the Health and Social Care Act 2012 that followed were predicated on the assumption that “individual creativity and innovation is best supported by competition”.^13^ Competition in healthcare services, which does not necessarily imply privitisation, was intended lead to greater patient demand for innovative treatments. The assumption was that this, in turn, would ensure that individuals live longer and healthier lives. However, a recent editorial on the impact of market-based reforms concluded that current research “offer[s] remarkably similar conclusions about the limited potential of markets in health and social care to deliver aspirations for improvements in both the quality and cost of care”.^14^ In contrast, preliminary findings from other studies featured in a recent Nuffield Trust report suggest cautionary optimism.^15^

Despite the lack of clarity, there continues to be a policy push towards competition in the NHS. Although the primary focus of the 2012 Act may have been increasing competition among healthcare providers such as acute hospitals, commissioning has also been affected, creating a fragmented healthcare landscape with commercial companies, not-for-profit agencies, social enterprises, voluntary sector bodies, commissioning support units, freelance consultants and public health professionals, all vying to improve and influence commissioning by supplying commissioners with information, advice and support. But some of these new providers need substantial help themselves. For example, the Nuffield Trust published guidance for voluntary organisations interested in offering commissioning support in November 2013.^16^

A single case study of a collaboration between a commercial provider and commissioning organisation has been reported in the literature. It found ‘strong relationships’ and ‘high levels of trust’ between the commissioners, commercial provider and healthcare provider.^17^ In addition, a survey with 172 responses from commissioners found good levels of satisfaction, with most rating their contracted commercial providers as ‘good’ or ‘excellent’.^18^ Yet the controversy around external providers persists, particularly commercial companies.

To help inform the debate, clarifying what external providers offer is of particular interest. Definitions of ‘knowledge’ and ‘knowledge exchange’ proliferate in the literature.^19^ In this paper, knowledge is defined as any tacit or explicit information, skill or expertise and ‘exchange’ is defined as reciprocal transfer. For example, the knowledge from commercial and not-for-profit companies under study included technical skills in deploying software tools and expertise in applying and interpreting data output. However, clients also had valuable knowledge to share, such as which local general practices would be most receptive to software tool deployment and how to modify the software to maximise its usability.

The aim of this study was to contribute to the debate about the use of external providers in the NHS by understanding how commissioners and external consultants work together, the processes of knowledge exchange and the perceived impact on commissioning decisions. This study includes data for contracts both predating and occurring contemporaneously with the implementation of the 2012 Health and Social Care Act.

## Methods

### Study design

We selected a mixed methods case study approach, as appropriate for exploratory questions in a real-life context, where there are few opportunities to control events and settings.^20^

### Case site selection

We recruited two commercial providers and one not-for-profit agency. The first external provider approached a competitor on our behalf, following ‘snowball’ sampling recruitment, which is an accepted feature of ethnographic research.^21^ This competitor became the second commercial provider under study. A not-for-profit company was approached and then recruited to contrast commercial and not-for-profit providers.

To preserve anonymity, we will use the term ‘external provider’ to mean both commercial and not-for-profit for the organisations studied. Using pseudonyms, the external providers were:

- Heron—a multinational with a suite of software tools and mixed expert UK/ non-UK staff, offering analytics and project management.
- Jackdaw—a small, international company offering one software tool.
- Swallow—a national company with a suite of software tools staffed largely by ex-NHS personnel offering analytical and commissioning expertise.

Each external provider was treated as a case. An additional case study was drawn from a subcontract within the Swallow data, making four external provider cases.

To access the views of NHS commissioners, we recruited four commissioning organisations that had contracts with at least one of the external providers. Using pseudonyms, these were:

- Carnford CCG—struggling financially, highly collaborative with its healthcare providers and reliant on the use of tools and the data produced from those tools to influence commissioning decisions.
- Deanshire CCG—relatively confident as a commissioning organisation, focused on governance, carrying out some innovative projects in partnership with commercial providers.
- Norchester CCG—financially challenged, emphasis on (ideally academic research) evidence-based policy making, piloting new ways of commissioning contracts, with substantial aid from commercial and not-for-profit providers.
- Penborough CCG—creating an integrated network of health and social care provision with a heavy emphasis on public involvement, historically extensive use of commercial and not-for-profit providers and freelance consultants.

At the close of fieldwork, we had eight case studies of the transactions between external providers and commissioning organisations. We were specifically interested in contracts with a significant knowledge exchange component at various stages, for example, beginning, middle and postcontract. Some commissioning organisations had contracts with more than one study provider, for example, Norchester worked with both Swallow and Jackdaw and some contracts included commissioning organisations other than Carnford, Deanshire, Norchester and Penborough.

### Data collection

We collected interview, observation and documentary data from February 2011 to May 2013, which was 9 months after the publication of *Liberating the NHS* the White Paper that led to the 2012 Health and Social Care Act. Data were collected by LW and EB, who are experienced qualitative researchers.

#### Interviews

For each external provider, we first interviewed senior leaders (Chief Executive or Directors). Through snowball sampling, we identified other candidates for interview with relevant knowledge or experience of the external provider and/or contract. For Swallow and Jackdaw, the research team independently approached candidates, usually by email. For Heron, interview requests from the research team were co-ordinated by a Heron consultant. For the commissioning organisations, we interviewed the lead NHS contact for the contract (usually identified by the external provider) and employed snowball sampling to identify other candidates, including lay representatives, local councillors, freelance and commercial consultants from other companies. Two NHS healthcare commissioners and one external consultant declined to be interviewed, while several others did not respond to requests.

Candidates were sent information before interviews and consent was obtained at interview. Following initial conversations with commissioners and external consultants, the research team devised a topic guide. It covered type of knowledge wanted by commissioners, information sources, how information was accessed and influenced decisions. The topic guide was revised as new questions emerged. Interviews were face to face or by telephone, depending on the preference of the participant and practicalities. Lasting 20–60 min, all interviews were recorded and transcribed by an external transcriber.

Data saturation was reached with larger external providers when over 15 external consultants were interviewed. As a small company, Jackdaw's consultants were all interviewed. Data saturation for commissioning organisations was reached when about half of the relevant stakeholders with direct experience of the contract were interviewed, although this proportion had not been predetermined. In total, we interviewed 92 participants including 47 NHS clients, 36 external consultants and 9 others (eg, freelance consultants, lay representative; table 1).

**Table 1:** Interview participants

Professional role	Number of participants
NHS	
Managerial commissioner	17
Clinical commissioner	15
Analyst	9
Other NHS	6
Total NHS	47
External	
Commercial/not-for-profit consultants	36
Freelance	3
Public health	4
Local authority	1
Lay representative	1
Total external	45
Total study	92

We conducted 25 observations of meetings between external consultants and their NHS clients, external provider and commissioner team meetings and training events. Permission was obtained verbally before attending events. Observation notes were taken with the help of an aide memoire based on the research questions and included details of participants, room layout, verbal exchanges and researcher reflections. Notes were typed up as soon as possible after the data were collected. All interview and observation participants were given pseudonyms. Meeting minutes, reports, website and marketing material, press releases and emails were collected and fed into the case summaries. These supplemented, confirmed and challenged emerging findings from interview and observation data.

### Data analysis

Our analysis, in common with much qualitative work, used deductive and inductive processes, and a similar approach has been described elsewhere in the literature.^22^ Initial analyses of the data were inductive, and used constant comparison to identify codes and compare these and emerging categories. This process was repeated and fed back into data collection (and further analysis) cycles.^23^ The study team met regularly to identify emerging themes, reflect on the research questions and suggest new questions for the fieldwork. Although not discussed explicitly in this paper, theories which the authors have previously engaged with about the ‘social life of information’,^24^ ‘communities of practice’,^25^ ‘mindlines’^26^ and ‘organisational sense-making’^27^ informed our analysis. Through reflective discussion among the team, we examined how these theories, as well as the initial research questions, deductively informed our analysis (eg, during discussion of one data item a team member noted knowledge transformation using ‘mindlines’ at which point the team discussed reasons for this, challenged it and explored cases that supported and refuted this assertion).

By May 2013, when fieldwork came to a close team members (EB, LW and AC) developed a coding framework based on these discussions and framed around the research questions. Using NVIVO software, EB and LW systematically coded cases and developed 20–50 page case summaries for each case structured around five domains. Four of these domains were deductively derived from the original research questions (external providers, knowledge accessed, knowledge transformation, benefits/disadvantages). The final domain (models of commissioning) emerged inductively from the analysis and surrounding discussions. Every member of the research team read these summaries independently and conducted cross-case analyses, identifying key themes common to the cases and searching for discrepant data. The team then met to finalise the agreed key themes.

### Challenges

Few previous studies have recruited commercial or not-for-profit consultants working in the NHS. Challenges included research governance, as external consultants moved freely and quickly around NHS organisations while researchers adhering to research governance could not. We wanted to shadow external consultants, but many had concerns about client sensitivities so we relied on observation of training events and larger meetings. Concerns were also expressed about patient data confidentiality, despite local R&D permissions. Nonetheless, the general enthusiasm and willingness of external consultants to participate in this study was noteworthy.

## Results

The core themes that emerged in determining what external providers offer were: (1) technical transfer (eg, software tool training, operation and application), (2) expertise (eg, knowledge and skills in project management and analytics) and (3) outsourcing (eg, taking over commissioning teams/units wholesale). They were also engaged for their ‘big picture’ perspective, potential to challenge local stakeholders, knowledge from international and national sources and new approaches to recurrent problems. The following vignettes, which sometimes depicted entire contracts and sometimes just one work stream, illustrated what external providers offered, how commissioners and external consultants worked together and their perceived impact. In selecting the vignettes, we chose one from each participating external provider where we had sufficient client-external provider accounts. In meeting the objectives of the study, the selected vignettes demonstrated a range of the ‘offers’ available.

### Vignette 1: A technical solution—but what is the problem?

The external provider in this vignette was imbued with ‘public sector values’, as the dissemination arm of an academic institution. Marketing a software tool to identify individuals at higher risk of using healthcare resources such as hospital beds, this external provider worked in partnership with other for and not-for-profit companies to reach clients, sometimes via academics.

It's often that the academicians (sic) through publications, through presentations and conferences and so on, that proves the [tool's] viability within a particular country or setting, and demonstrates its value. And then the government gets—you know—it gets their attention. (External consultant, Katie)

After a 6-month needs assessment, this software tool was selected by a team of senior information managers acting on behalf of a consortium of commissioning organisations that wanted to “club together and think about how they could do commissioning in a more effective way” (NHS information manager, Shauna). However, once the tool was fully deployed (about 3 years after the original needs assessment exercise), the procurement team realised that the basic training for the tool offered by an intermediary external provider was insufficient. They contracted the tool developers directly to procure advanced training.

The training by the tool developers was delivered by experts from North and South America, with little knowledge of the NHS, to seven NHS clients of diverse backgrounds (analytics, primary care commissioning, project management) via webinars. The training was almost entirely technical, which was appreciated by healthcare analysts who confidently applied their new knowledge in novel ways, for example, using the software tool to allocate general practice budgets. But technical knowledge alone was insufficient for some NHS clients. For example, a primary care commissioner talked about how they had not ‘chosen’ but were ‘given’ the tool, and then had to find an application. Another client talked about the difficulties in contextualising tool outputs to local circumstances without a data interpreter and a clear strategy from senior NHS managers about how the tool should be used.

I think what would be really useful is somebody from [external provider] to work with the strategic [commissioning] lead and maybe myself to actually think about the best way to use it to get the maximum results. So do we just look at COPD? Do we look at diabetes? Is there something that we can do with the tool that would give us a really quick win? (NHS project manager, Kourtney)

Overall at the time of fieldwork, use of this tool had had limited impact on informing commissioning, although it was early days as training was ongoing. The lessons from this vignette are that technical ‘solutions’ can only solve clearly identified and recognised problems. Moreover, translators who can interpret data outputs are necessary and maximising those outputs relies on external consultants, analysts and commissioners working together.

### Vignette 2: A new approach to a recurrent challenge

The external provider in the second vignette also offered technical transfer through software tools. With one tool, clinical reviewers compared patients’ notes to a set of standards based on expert consensus on ‘best place of care’ (ie, hospital or community based). Patients either ‘qualified’ to be in their current setting or did not. At the instigation of commissioners, two audits using this tool were carried out for an acute trust, as a way of identifying unnecessary hospital admissions.

The first audit was entirely conducted by external consultants in autumn 2010 and was described as a ‘disaster’ (Medical Director, Hugh). Many patients were identified as ‘not qualifying’, a finding contested by the hospital, which placed further strain on already difficult relationships between the commissioners and hospital. Nine months later, after the shortcomings of the first audit had been agreed, a second audit took place in summer 2011, carried out this time by five local reviewers from the hospital, community provider and commissioning agency. Local reviewers initially learnt how to use the tool during a 2-day training session and then that learning was augmented and consolidated through experiential application of the tool during data collection, with external consultants on hand acting in an advisory capacity. Interestingly, the proportion of patients ‘not qualifying’ in the second audit (24%) was almost the same as the first audit (28%), but local ownership meant that the second audit results were more readily accepted.

The NHS clients found the second audit ‘very useful’ (NHS information manager, Joan), but not because the tool gave much insight into unnecessary hospital admissions. Rather through joint data collection with daily de-briefing sessions chaired by the hospital medical director, professionals from different care sectors learnt more about each other's norms and challenges and developed better working relationships. The hospital team also learnt to think differently about ways to reduce hospital admissions (ie, from the perspective of where the patient is best placed).

I think the whole question of looking at admissions and what was required, and what services could be put around it, is one that is so obvious that actually we weren't thinking about it…And so by modifying that concept I think we will learn a lot and gain a lot. So I think they [external provider] did bring that. (Medical director, Hugh)

Further audits using the same method (but not the software tool) were conducted in other hospital wards, but the external provider was not involved. Several months after the second audit, we received an email stating that the results had not fed into any commissioning decisions, but that the ensuing local relationships were highly valued. The lesson from this vignette was that where possible, external consultants could helpfully ensure that the work is conducted by clients, so that the clients take ownership and skills are more easily transferred.

### Vignette 3: ‘Going from good to great’

In addition to contracting external providers for their technical offer, as illustrated in the previous two vignettes, external consultants were also engaged for their expertise in project management. The NHS commissioning organisation involved in this vignette was “trying to go from good to great as a commissioner” (Carol, NHS commissioning manager), so one of the numerous work streams was to carry out a set of activities to help their NHS clients prepare for a ‘World Class Commissioning assurance day’.

As part of this process, the external consultants carried out a ‘gap analysis’, based on the World Class Commissioning competencies, where they challenged their NHS clients to “demonstrate that you actually do that. Give me the tangible evidence” (Helen, external consultant). Other activities included identifying experts in commissioning to visit the client site, setting up visits to other NHS commissioning organisations, engaging local clinicians and providing project management training and tools. The external consultants sought to complement the client's strengths, and a commissioning manager spoke about some of the processes used:

They often brought people in, drafted people in from [North America] to talk to us about ideas that we were having. So quite often we'd have ideas or they would suggest ideas to us about what we could do locally, and they would expand and build on that, and come back with a rounder package, which we would then test out. (Sarah, NHS commissioning manager)

The culture within the NHS client organisation prioritised collaboration, innovation, transparency and engagement. This may have fostered the strong relationship they developed with the external consultants, who commented on how this had gone beyond developments with other NHS clients, where external consultants were sometimes perceived as a threat. The consultants described the client commissioners in terms of Belbin's team roles, which the external team deliberately complemented.^28^

So they were the plants and shapers, but they weren't the completer finishers. I would say that that was evident when we were working with them, that they had a huge amount of ideas. Lots of shaping, lots of meetings, huge meeting culture, and then the actual discipline of completing it and measuring it was not there. (Patricia, external consultant)

The main challenges raised by both sides were those of defining what work was needed, and how to ensure that work from the World Class Commissioning work stream and others remained relevant.

Over the two year period the world changed around us, so we ended up having to reset what it was that we needed from them several times. And I'm sure you can appreciate that that takes—it's a little like a juggernaut, isn't it?—it takes turning around and renegotiating, for them and us, of what was needed, and finding out that something different was needed, and putting that into place, meant things stalled several times along the way. (Carol, NHS commissioning manager)

The NHS clients achieved their goal by being rated among the top five English commissioning organisations. Most NHS participants were pleased with this result, although subsequently a few queried whether this had been worth the cost. The external consultants also valued learning from their NHS clients, as previously they had not helped clients with World Class Commissioning assurance processes. The lesson in this vignette is that knowledge exchange is possible when client organisations are ready and willing to work with external providers who, in turn, are adaptable and complement (rather than replace or duplicate) the commissioners’ skills.

### Vignette 4: ‘Data-driven’ commissioning

Although external providers in this study mainly offered technical solutions and expertise, there was one example where commissioning had been completely outsourced. The external provider managed all aspects of the contracts of a group of hospitals worth over £100 million, which were described as “very expensive and quite difficult to control” (Joel, external consultant).

The external provider perceived the NHS as driven by politics and people rather than by data, whereas their own ethos was to “use data to drive decision making” (Kristen, external consultant). The external team consisted of a programme manager, administrators and ‘lots of analysts’, who undertook ‘forensic investigation of the data’, mainly by finding errors in coding leading to overcharging (Joel, external consultant). Nurses, with essential clinical knowledge, were also placed in hospitals to verify patient notes against invoices, as commissioners had limited ways of checking the accuracy of claims. The approach the external consultants took with healthcare providers was confrontational.

[We said]…“If you don't supply us with this data, we can't validate our patient activity, therefore we are not going to pay for it.” So [a] slight—at times, very—antagonistic approach. (Dennis, external consultant)

In an attempt to reduce hostility, a NHS commissioner was seconded for 1 year to improve relationships between the external provider, the NHS hospitals and local commissioning organisations mid-contract. During fieldwork, several participants noted that relationships were better, partly due to this intervention.

Analytical expertise and good quality data were highly valued by this external provider to inform decision-making. The ‘standard’ team they offered consisted of an analyst, project manager and clinical lead in contrast to the NHS, where analysts, commissioning managers and clinicians tended to work separately in silos. A NHS client said this analytical support was vital and that the external provider did “the basics really well” (Jacob, NHS commissioning manager). This resulted in savings estimated as over a million pounds.

Initially the draft contract had included a knowledge transfer strategy so that a NHS team could develop these analytical skills. But this clause was eliminated by the NHS client to reduce contract costs. This contract was repeatedly renewed. As a result, by 2019, the external provider will have operated this outsourced commissioning service for 10 years with no mechanisms in place to develop skills within the NHS. The lesson from this vignette is that if clients and external providers do not agree knowledge transfer strategies within the contract to the NHS client organisation or other external providers such as commissioning support units, the client is likely to end up reliant on support from one external provider long term. This creates a monopoly, which is at odds with both the competitive thrust of the 2012 Health and Social Care Act and which also, importantly, undermines the influence of local clinical intelligence that the government has stated should be at the heart of commissioning.

## Discussion

### Principal findings

External provider involvement was intended to improve the quality of commissioning. To achieve this, external providers offered technical applications, expertise and outsourcing. The impact of the contracts illustrated in these vignettes was shaped by the original objectives of the contracts and the expectations and ability of external providers and client organisations to meet those objectives (which may have been overoptimistic). We recognise that the ‘success’ or failure of these contracts is multidimensional and can be understood in the short and long term. With this in mind, we suggest that these vignettes show that external providers were only partly successful in improving the perceived quality of commissioning, largely because the knowledge exchange interactions between external providers and NHS clients were limited. In fact, only in vignette 3 was there substantial genuine knowledge exchange, with both sides receiving benefits, as in the other vignettes knowledge went just one way (ie, external provider to client). The use of external providers proved problematic in several ways.

Vignette 1 illustrated that access to a software tool and technical training was inadequate; external providers needed to supply translators who could interpret the data, work with clients to contextualise outputs and help identify ways to use the outputs to inform commissioning decisions. Without this, the software tools did not address genuine problems currently being experienced, because of changes since initial procurement and insufficient consultation with client operational staff. There was also a split between the senior management agenda and those expected to operate or be informed by the tools. Contracts with external providers co-produced by all the actively interested parties may have a greater chance of success. If not, the tools can become a time-consuming problem in their own right.

Vignette 2 emphasised the importance of clients undertaking the work themselves, such as audit data collection, rather than relying on external providers. But often NHS participants reported limited time or capacity, especially following the launch of *Liberating the NHS*, which led to the departure of many experienced commissioning staff. Transferring skills and knowledge to clients may appear to undercut future procurement of external providers, but conversely may increase trust and perceived usefulness, which could improve the prospects of repeat business. This vignette highlighted another key point, mainly that the impact from contracting the external provider had unanticipated benefits such as adoption of an innovative method (but not the product itself) and the serendipitous mending of previously fractured relationships among local healthcare organisations that needed to work together. In vignette 2, client participants found these outcomes more useful than the direct input of the external provider, which was described as of little value.

Vignette 3 was an example of what commissioners and external consultants could achieve together—if healthcare clients at all levels were genuinely willing and ready (which may not be the case). The external consultants adapted their expectations to fit clients’ reality and negotiated mutually acceptable understandings and timeframes. Moreover, the external consultants complemented their NHS clients by matching consultant ‘completer/finishers’ to client ‘blue sky thinkers’. In allocating external consultants to clients, these less obvious characteristics received careful thought during procurement. The clients also learnt useful new skills such as ways of measuring the impact of their commissioning activities. Overall, this contract appeared to meet clients’ expectations.

Vignette 4 was undoubtedly a short-term success in financial savings to the NHS, but not in longer term improvement in the perceived quality of commissioning among the NHS clients. This finding cautions both external providers and their NHS clients to value and make provision for explicit knowledge transfer mechanisms, as the NHS clients ended up dependent on the external consultants’ increasing monopoly of skills. The potential benefits through skilling local staff were not realised and longer term the role of local clinical intelligence was diminished. Given that the success of this contract was largely due to the significant input of analysts, finding ways of cross-pollinating analytical, clinical and managerial expertise through the use of ‘standard’ teams consisting of professionals from each group may help bring about more ‘data-driven’ commissioning in the NHS, reducing dependency on external providers.

### Strengths and weaknesses

This is the largest study of commercial and not-for-profit providers and healthcare commissioners following the 2012 Health and Social Care Act. These external providers permitted substantial access and provided a comprehensive view of their work, although we note that perspectives from NHS clients, especially operational analysts and commissioners, were harder to obtain. We recognise that entering the field via the external provider may have affected NHS recruitment and we would have liked to recruit more ‘negative’ cases from one external provider, who steered us away from less successful contracts. However, ample data were collected, both positive and negative, to create coherent case studies, which provide conclusions based on carefully collected and systematically analysed data.

### Relevance of study with regard to wider literature

There is scant literature on use of external providers in the NHS. A study published before the Health and Social Care Act 2012 concluded that commissioners did not always use external support from commercial providers to its full potential, which our study confirms.^18^ We found factors contributing to success included building effective working relationships, which were partial in vignette 1 and absent in vignettes 2 (hospital audit) and 4 (data-driven commissioning). The importance of trust and good working relationships was also identified in a post-2012, single case study of collaboration between clinical commissioners and external providers^17^ and in a recent study of commissioning support units.^29^ In fact, this latter study concluded that good quality internal relationships are so important to commissioners, that in commissioners’ determination to forge these links, they are bringing commissioning support analysts, who were their former commissioning colleagues before the 2012 Health and Social Care Act, back into CCGs. This directly challenges current governmental policy on competition.

Although the literature on use of external consultants in the English NHS is sparse, an impressive, instructive body of literature exists on the use of commercial consultants in the private sector. For example, a study of commercial consultants in the Canadian telecommunications industry found that the single most important factor of success was the willingness of commercial companies to adapt to ‘client readiness’,^30^ which was evident in vignette 3 where commissioners at all levels were highly motivated to improve their World Class Commissioning rating. Another Canadian management academic put forward six propositions for successful engagement including a clear agreement concerning requirements and expectations, which was missing in vignettes 1 and 2 where the NHS operational staff did not co-produce or contribute to the contract at the procurement stage. A further marker of success was a good fit between consultant and client, including consultant type,^31^ which was present in vignette 3 (eg, allocating ‘completer/finishers’). However, despite the prevalence of this literature, and other relevant studies, once again we note that the findings of research have made a limited impact on policy and practice within public services.^32^ As contracts with external consultants become more widespread, drawing this literature to the attention of both external providers and healthcare commissioners who are using external support will become more imperative.

## Conclusion

A major goal of the Health and Social Care Act 2012 was to introduce multiple types of external providers to increase competition with the assumption that this leads to improved quality of commissioning. This assumption is problematic, as the impact of competition on healthcare has yet to be clarified, even with regard to service provision, which is where this embryonic research field has focused to date.^14^ ^15^ Much less is known about the impact of competition on commissioning. But even if competition were likely to improve the quality of commissioning, our study suggests that the right elements may not be in place to optimise any such benefits.

Several features were crucial to achieving positive impacts from involving external providers, such as a clearly agreed problem of relevance and importance to both operational and managerial staff and co-produced solutions. This indicated genuine client ‘readiness’ to work with external providers. Other characteristics were continual reassessment of the problem (and proposed solution) and local staff taking responsibility for undertaking the work to learn new skills, instead of relying largely on external consultants. If the contract involved information provision, external providers needed to supply not only technical solutions, but also skills in interpretation with locally contextualised strategies to inform commissioning, developed in genuine partnership with the right NHS staff. One way of improving the impact of data on commissioning might be for commissioners to adopt the model from the external provider in vignette 4 by using integrated internal teams of clinicians, analysts and managers to cross-fertilise expertise. Without these elements, the use of external providers appears to have only sporadic benefits of limited value for commissioning.

However, this raises a dilemma. If local expertise is essential for high-quality commissioning, then employing a non-local external commercial or not-for-profit provider to develop and supply such expertise puts the contracting organisation in a vulnerable position, as the contracting organisation becomes increasingly dependent on the external provider (as illustrated by vignette 4). This is likely to worsen over time. But developing the expertise in-house does not solve the problem either, unless there is a plan to maintain that expertise to be resilient to shocks such as reorganisations and departures of key personnel.

The NHS is increasingly contracting with external providers to help with the commissioning process and the current government is encouraging this, while at the same time wanting to ensure that local clinicians and their patients have primacy in the decision-making. That being so, then, at the minimum, knowledge exchange strategies need to be enshrined explicitly in such contracts in order to optimise commissioning by developing and enhancing local skills. Both NHS clients and external providers have an obligation to NHS patients to ensure that the potential for knowledge exchange is fully exploited.

## References

[1] Newman M, Bangpan M, Kalra N Commissioning in health, education and social care models, research bibliography and in-depth review of joint commissioning between health and social care agencies. London: EPPI Centre Institute of Education University of London, 2012.

[2] Øvretveit J Purchasing for health: a multi-disciplinary introduction to the theory and practice of commissioning. Buckingham: Open University Press, 1995.

[3] Department of Health. Health Reform in England: update and commissioning framework 2006.

[4] Checkland K, Snow S, McDermott I Management practice in primary care organisations: the roles and behaviours of middle managers and GPs: final report. NIHR Service Delivery and Organisation Programme, 2011.

[5] Smith J, Shaw S, Porter A Commissioning high quality care for people with long term conditions: an action research study. NIHR Service Delivery and Organisation Programme, 2013.

[6] Ham C World class commissioning: a health policy chimera? J Health Serv Res Policy 2008;13:116–21. 10.1258/jhsrp.2008.007177 18416918

[7] Department of Health. World Class Commissioning. London: Department of Health, 2007.

[8] Mooney H HSJ commissioning supplement: an in-depth look at FESC. Health Serv J 2007.

[9] Welikala J Private firms and local authorities in running to provide commissioning support. Health Serv J 2014.

[10] NHS England. Lead provider framework. Secondary Lead provider framework 2014 http://www.england.nhs.uk/ourwork/commissioning/comm-supp/ld-prov-frwrk/

[11] House of Commons Select Committee. The use of management consultants by the NHS and the Department of Health: fifth report of the 2008–2009 session. London: The Stationery Office, 2009.

[12] Department of Health. Liberating the NHS. London: Department of Health, 2010.

[13] Langsley A Andrew Lansley: competition is critical for NHS reform. Health Serv J 2012. 22468455

[14] Dickinson H, Shaw S, Glasby J The limits of market-based reforms. BMC Health Serv Res 2012;13 (Supp 1):11 10.1186/1472-6963-13-S1-I1 PMC3663651 23734949

[15] Charlesworth A, Kelly E Competition in UK health care: reflections from an expert workshop. London: Nuffield Trust, 2013.

[16] Holder H Role of the voluntary sector in providing commissioning support. London: Nuffield Trust, 2013.

[17] Chambers N, Sheaff R, Mahon A The practice of commissioning healthcare from a private provider: learning from an in-depth case study. BMC Health Serv Res 2013;13 (Suppl 1):S4 10.1186/1472-6963-13-S1-S4 PMC3663660 23735082

[18] Naylor C, Goodwin N The use of external consultants by NHS commissioners in England: what lessons can be drawn for GP commissioning? J Health Serv Res Policy 2011;16:153–60. 10.1258/jhsrp.2010.010081 21708918

[19] Contandriopoulos D, Lemire M, Denis JL Knowledge exchange processes in organizations and policy arenas: a narrative systematic review of the literature. Milbank Q 2010;88:444–83. 10.1111/j.1468-0009.2010.00608.x 21166865 PMC3037172

[20] Yin R Case study research. 3rd edn London: Sage, 2002.

[21] Hammersley M, Atkinson P Ethnography: principles and practice. London: Routledge, 1995.

[22] Fereday J, Muir-Cochrane E Demonstrating rigor using thematic analysis: a hybrid approach of inductive and deductive coding and theme development. Int J Qual Methods 2006;5:80–92.

[23] Patton M Qualitative research and evaluation methods. London: Sage, 2002.

[24] Brown J, Duguid P The social life of information. Boston, MA: Harvard Business School Press, 2000.

[25] Wenger E Communities of practice: leaning, meaning and identity. New York: Cambridge University Press, 1998.

[26] Gabbay J, le May A Practice-based evidence for healthcare. Oxford: Routledge, 2011.

[27] Weick K Making sense of the organisation. Oxford: Blackwell Business, 2001.

[28] Belbin RM Management teams: why they succeed or fail. 3rd edn Oxford: Butterworth-Heinemann, 2010.

[29] Petsoulas C, Allen P, Checkland K Views of NHS commissioners on commissioning support provision. Evidence from a qualitative study examining the early development of clinical commissioning groups in England. BMJ Open 2014;4:e005970 10.1136/bmjopen-2014-005970 PMC4202006 25320000

[30] Appelbaum S, Steed J The critical success factors in the client-consulting relationship. J Manag Dev 2005;24:68–93. 10.1108/02621710510572362

[31] MacLachline R Factors for consulting engagement success. Manag Decis 1999;37:394–402. 10.1108/00251749910274162

[32] Nutley S, Walter I, Davies H Using evidence: how research can inform public services. Bristol: The Policy Press, 2007.

